# RB1 aberrations predict outcomes of immune checkpoint inhibitor combination therapy in NSCLC

**DOI:** 10.3389/fonc.2023.1172728

**Published:** 2023-06-27

**Authors:** Qian Wang, Tao Yu, Zi-Hao Ke, Fu-Feng Wang, Jia-Ni Yin, Yang Shao, Kai-Hua Lu

**Affiliations:** ^1^ Department of Oncology, the First Affiliated Hospital of Nanjing Medical University, Nanjing, Jiangsu, China; ^2^ Geneseeq Research Institute, Nanjing Geneseeq Technology Inc., Nanjing, Jiangsu, China; ^3^ School of Public Health, Nanjing Medical University, Nanjing, Jiangsu, China

**Keywords:** immune checkpoint inhibitors, RB1, non-small cell lung cancer, next-generation sequencing, cell cycle, chromosomal instability, combination immunotherapy

## Abstract

**Introduction:**

Immune checkpoint inhibitors (ICI) have changed the treatment of non-small cell lung cancer (NSCLC). Furthermore, compared with monotherapy, ICI combination therapy had better efficacy and partly different mechanism. Therefore, we aim to investigate and improve biomarkers specialized for ICI combination therapy.

**Methods:**

We enrolled 53 NSCLC patients treated with ICI combination therapy and collected their tissue and plasma samples to perform next-generation sequencing (NGS) with a 425-gene panel.

**Results:**

The line of treatment was the only clinical factor significantly affecting objective response rate (ORR) and progression-free survival (PFS). Surprisingly, classical markers PD-L1 and TMB only had limited predictive values in the ICI combination therapy. Instead, we found RB1 mutation was significantly associated with prognosis. Patients with mutated RB1 had shorter PFS than those with wild RB1 (134d vs 219d, p=0.018). Subsequent analysis showed the RB1 related mutated cell cycle and chromosomal instability were also deleterious to prognosis (103d vs 411d, p<0.001; 138d vs 505d, p=0.018). Additionally, patients with more circulating tumor DNA (ctDNA) had significantly shorter PFS (41d vs 194d, p=0.0043).

**Conclusion:**

This study identified that NSCLC patients with mutated RB1 were less sensitive to ICI combination therapy. RB1 mutations and following cell cycle abnormalities and chromosomal instability can potentially guide clinical management.

## Introduction

Immune checkpoint inhibitors (ICIs) have been used to treat a wide variety of malignancies with remarkable success. PD-1/PD-L1 monoclonal antibodies have shown significant clinical benefits for late-stage non-small lung cancer (NSCLC) (1). Combination therapy with ICI could further produce promising antitumor activities and provide more survival benefits for patients with advanced NSCLC (2). Combined chemotherapy or anti-angiogenic agents can efficiently circumvent tumor resistance to ICI (3). Therefore, combination immunotherapy is recommended for NSCLC patients without driver mutations. However, the number of patients who benefit from the combined regimens remains limited. Resistance to combined treatments also develops invariably. It remains challenging to predict the response to ICI combination therapy and monitor disease progression. The identification of novel biomarkers is expected to enhance the efficacy of ICI combination regimens for NSCLC.

PD-L1 expression and tumor mutational burden (TMB) are classical predictive markers for screening patients sensitive to ICI (3). PD-L1 is measured using immunohistochemistry (IHC), and TMB is usually calculated using large next-generation sequencing (NGS) panels. Clinical guidelines recommend anti-PD-L1 monotherapy for patients with > 1% PD-L1 expression (4). TMB has also been shown to be associated with immunotherapy (5). However, several shortcomings have limited the application of these biomarkers. First, sampling sufficient tissue from patients with advanced NSCLC for detection is challenging. Samples with spatiotemporal heterogeneity may not accurately reflect immunogenicity (6). Patients with low PD-L1 expression could also benefit from immunotherapy (7), addressing the imperfections of currently available markers and the need to identify complementary markers. Additionally, the predictive value of TMB in solid tumors without sufficient T-cell infiltration, such as lung squamous cell cancer (SCC), is limited. The inclusion of driver mutations may also bias the calculation of TMB (8). The association between TMB and efficacy has not been verified in the KEYNOTE 189 trial (9). Moreover, combined regimens may change the immunogenicity reducing value of the existing biomarkers. Therefore, more precise predictive indicators for ICI combination treatment stratification are required.

It has been gradually established that gene mutation signatures could serve as a good complement for prediction (10). Multiple orthogonal gene alterations have been considered in precision combination regimens. For example, TP53-mutated lung cancers have significantly higher levels of antitumor immune signatures than TP53-wildtype cancers (11). Mutated KRAS modestly increases responsiveness to immunotherapy (12), whereas mutated STK11 or PIK3CA is associated with a lack of benefit (10, 13). Moreover, several genetic mutations, such as MDM2 amplification and EGFR aberrations, can indicate hyperprogression after immunotherapy (14). These results suggest that gene mutations are possible predictors of outcomes of ICI combination therapy.

Consequently, we collected samples from NSCLC patients who received ICI combination therapy and conducted a retrospective study to explore the predictive value of gene mutations. NGS was used to determine the frequency of mutations (10). We comprehensively examined the mutation characteristics, pathway abnormalities, and chromosomal instability (CIN) in NSCLC and analyzed their association with the objective response rate (ORR) and progression-free survival (PFS). Additionally, blood samples can provide additional information for clinical decisions. High levels of circulating tumor DNA (ctDNA) are associated with poor survival (15). Maximum somatic allele frequency (MSAF) was used to determine the amount of ctDNA. MSAF is the maximum allele frequency of all somatic mutations identified per sample using NGS (16). MSAF can complement blood TMB (bTMB) to distinguish NSCLC patients with or without prognostic benefits from ICI (17). The performance of MSAF and bTMB in the baseline plasma samples was also investigated for efficacy prediction.

## Materials and methods

### Patients and response assessment

We retrospectively collected 67 late-period primary NSCLC patients who received immuno-oncology immunotherapy at Jiangsu Province Hospital between November 2017 and February 2022, and 14 patients were excluded due to mismatched stage and pathological type. The median follow-up period was 190 days.

Responses were assessed using the RECIST version 1.1. Progression-free survival (PFS) was defined as the time from the beginning of treatment to disease progression (PD) or death. The objective response rate (ORR) was defined as the sum of the complete response (CR) and partial response (PR) divided by the number of patients.

### DNA extraction and targeted NGS

Formalin-fixed paraffin-embedded (FFPE) tumor samples obtained through biopsy or surgical excision were collected. Samples with ≥20% tumor cell content were included in the study. 8-10 ml of Peripheral blood was collected in EDTA-coated tubes (BD Biosciences) and centrifuged at 1800 g for 10 min within 2h of collection to separate the plasma and white blood cells. Plasma was isolated for the extraction of circulating cell-free DNA (cfDNA) and white blood cell sediments were used for genomic DNA extraction as germline controls.

Genomic DNA from FFPE sections and whole blood control samples was extracted using the QIAamp DNA FFPE Tissue kit and DNeasy Blood and Tissue Kit (Qiagen, USA), respectively. cfDNA from the plasma was purified using the Circulating Nucleic Acid Kit (Qiagen, USA) following the manufacturer’s protocol.

Customized xGen lockdown probes (Integrated DNA Technologies) targeting 425 cancer-relevant genes (Geneseeq) were used for hybridization enrichment. The target-enriched library was then sequenced on the HiSeq4000 NGS platform (Illumina) with a mean coverage depth of 1000x for tumor tissue samples, 3000x for cfDNA samples and 100x for matched normal blood control samples, following the manufacturer’s instructions.

### Sequence alignment and data processing

Base calling was performed on bcl2fastq V.2.16.0.10 (Illumina) to generate sequence reads in FASTQ format (Illumina 1.8+encoding). Quality control was performed using Trimmomatic software. High-quality reads were mapped to the human genome (hg19, GRCh37 Genome Reference Consortium Human Reference 37) using Burrows-Wheeler Aligner (BWA) V.0.7.12 with Burrows-Wheeler Aligner’s maximal exact matches (BWA-MEM) algorithm and default parameters to create SAM files. Picard V.1.119 was used to convert SAM files to compressed BAM files, which were then sorted according to chromosome coordinates. The Genome Analysis Toolkit (GATK, V.3.4–0) was used to locally realign the BAM files at intervals with insertions/deletions (indels) mismatches and recalibrate the base quality scores of reads in the BAM files.

### Mutation calling, CNV, CIS, TMB

Single-nucleotide variants (SNVs) and indels were identified using VarScan2, with a minimum variant allele frequency at 0.01 for tissue and 0.001 for cfDNA. SNVs and indels were further filtered with the following parameters: (1) minimum mean dedup depth=30X (tissue and blood) and 600X(cfDNA); (2) minimum base quality=15, (3) minimum variant supporting reads=3, (4) variant supporting reads mapped to both strands, (5) strand bias no greater than 10%, (6) if present in >1% population frequency in the 1000 g or ExAC database and (7) through an internally collected list of recurrent sequencing errors using a normal pool of 100 samples. ANNOVAR was used to annotate mutations by variant type (18), dbSNP ID, clinical significance, and protein impact prediction using SIFT and PolyPhen (19, 20). Germline mutations were filtered out by comparing them with patients’ whole blood controls. Copy number variations (CNVs) were analyzed with CNVkit.14 depth ratios above 2.0 (tissue) or 1.6 (cfDNA) and < 0.6 were considered as CNV gain and CNV loss, respectively. The average proportion of the genome with aberrant (log2 depth ratio >0.2 or <−0.2) copy number, weighted on each of the 22 autosomal chromosomes, was estimated as the chromosomal instability score (CIS). TMB was calculated by summing all base substitutions and indels in the coding region of targeted genes, including synonymous alterations to reduce sampling noise and excluding known driver mutations as they are over-represented in the panel, as previously described (21).

### Pathway analysis

For cell cycle pathway analysis, genes related to cell cycle pathways were referred to in a previous article published in 2018 (22). Patients harboring one or more mutated genes were considered positive.

### Immunohistochemistry

PD-L1 expression was determined using the Dako PD-L1 IHC 22C3 pharmDx kit (Agilent Technologies) in combination with the Dako Autostainer Link 48 system (Agilent Technologies). PD-L1 expression was evaluated using the tumor proportion score (TPS), and a TPS ≥ 1% was defined as positive.

### Statistical analysis

For survival analysis, Kaplan-Meier curves were compared using the log-rank test, and hazard ratios (HRs) with 95% CIs were calculated using the Cox proportional hazards model. The reverse Kaplan-Meier method was used to calculate the median follow-up time. Fisher’s exact test was used for intergroup comparisons as needed, and the p value was calculated using a two-tailed test. All statistical analyses were performed using R version 3.5.0.

## Results

### Clinical information of enrolled NSCLC patients

**Figure 1:**
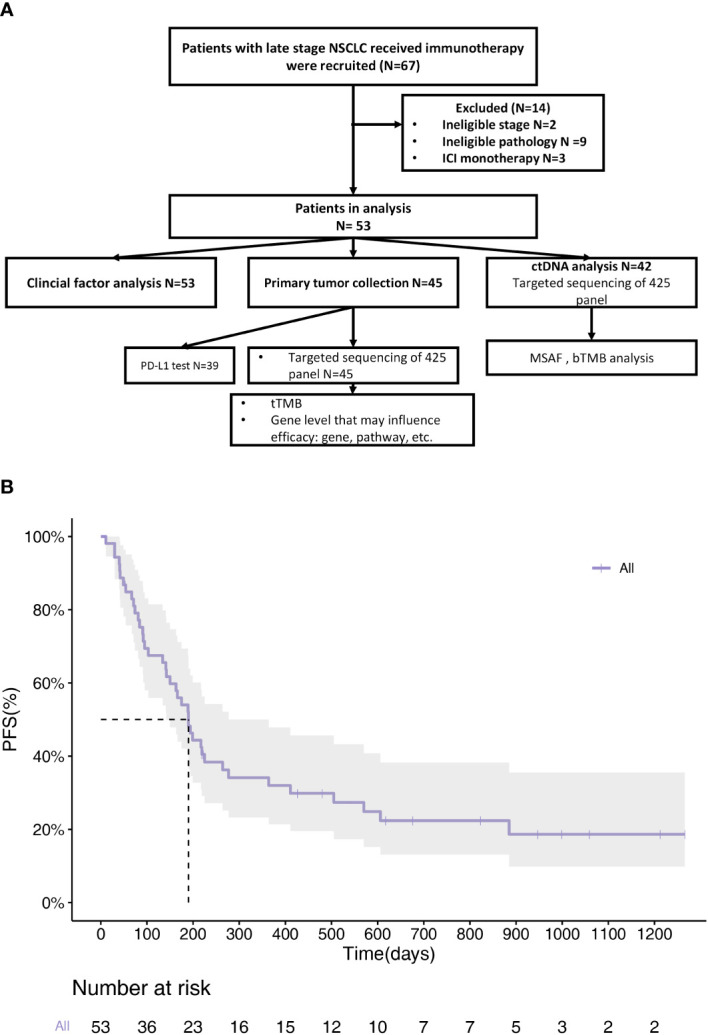
Clinical information of enrolled NSCLC patients. **(A)** Flow diagram of articles identified, included and excluded. A total of 67 patients were enrolled. 45 patients provided tissue samples and 42 provided blood samples for NGS. **(B)** PFS Kaplan-Meier survival curves of all patients. mPFS=190d (95%CI 142d-364d).

This study recruited 67 patients with NSCLC at JiangSu Province Hospital between November 2017 and February 2022. A schematic flowchart of the study is presented in **Figure 1A**. Fourteen patients with an ineligible pathology or monotherapy were excluded from the study. Finally, 53 patients with NSCLC were included in this study. The overall ORR was 39.6%, with 21 patients achieving PR and no patients achieving CR. The median PFS (mPFS) was 190 days (range 142-364) (**Figure 1B**). The clinical information of the enrolled patients is summarized in **Table 1**. The median PFS (mPFS) was 190 days (95%CI 142-364). The patients included 36 males and 17 females. 38% of the patients had a history of smoking. The main histological type was adenocarcinoma (ADC), which accounted for 70% of cases. Patients were staged according to the 8th edition of the tumor‐node‐metastasis (TNM) classification system. 79% of the patients had stage IV disease. 72% of the patients received first-line treatment. A total of 53 patients who had received combination treatment with immunotherapy were enrolled in the study. Among them, the majority had received immunotherapy combined with chemotherapy (n=31, 59%), the remaining had received immunotherapy combined with chemotherapy and anti-angiogenic (n=15, 28%), with anti-angiogenic (n=5, 9%), with targeted therapy (n=2, 4%). No association between the types of combination treatment with outcome was found.

**Table 1:** Baseline clinical characteristics of the study population.

Clinical information	All patients (N =53)
Median age (range)	69 (41-80)
Sex - No. (%)	
Male	36 (68%)
Female	17(32%)
Smoking history - No. (%)	
Yes	20 (38%)
No	31 (58%)
Unknown	2 (4%)
Cancer type - No. (%)	
ADC	37 (70%)
SCC	14 (26%)
Others	2 (4%)
Stage - No. (%)	
III	11 (21%)
IV	42 (79%)
First-line - No. (%)	
Yes	38 (72%)
No	15 (28%)
Treatment - No. (%)	
Anti-PD-(L)1 and Chemo	31 (59%)
Anti-PD-(L)1, Anti-angiogenic and Chemo	15 (28%)
Anti-PD-(L)1 and Anti-angiogenic	5 (9%)
Others*	2 (4%)

*Anti-PD-(L)1 and Targeted therapy N =1; Anti-PD(L)1, Anti-angiogenic and Targeted therapy n =1.

### Outcomes of NSCLC patients receiving ICI combination immunotherapy

**Figure 2:**
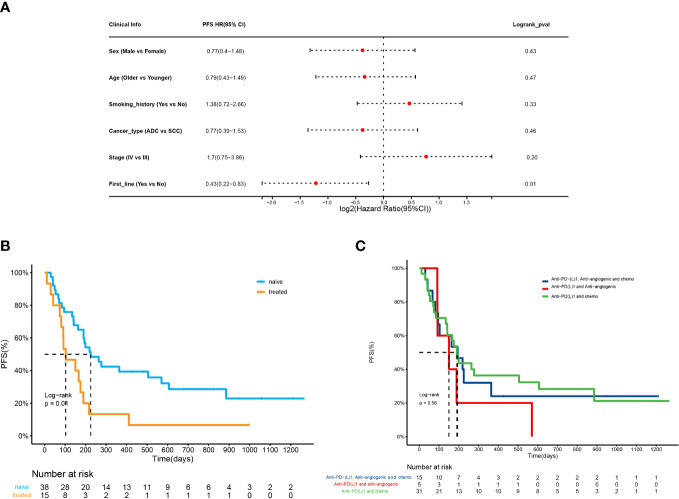
Outcomes of NSCLC patients receiving ICI combination immunotherapy. **(A)** Forest plot for 53 patients undergoing ICI combination therapy. The vertical line represents the hazard ratio (HR) of 0. **(B)** PFS analysis of patients receiving the first line treatment (naive) versus second- and late-line treatment (treated) (225d vs 103d, HR=0.43(0.22-0.83), p=0.01). **(C)** PFS analysis of patients receiving ICI plus chemotherapy, ICI plus anti-angiogenic agents, ICI plus chemotherapy and anti-angiogenic agents, and ICI monotherapy (p=0.56).

We analyzed the ORR and PFS to determine the efficacy of ICI combination therapy. The correlation between the clinical variables and ORR or PFS was also evaluated. The results showed that only the line of therapy was significantly associated with ORR and PFS (**Table S1**; **Figure 2A**). Patients receiving first-line treatment had a relatively higher ORR than patients receiving second- and late-line treatment (50.0% vs. 13.3%, p=0.03). The first-line treatment was a protective factor of PFS, whose HR was 0.43 (95% CI:0.22-0.83). In addition, ICI plus anti-angiogenic agents and chemotherapy tended to achieve the highest ORR (n=8, 53%). The ORRs of ICI plus chemotherapy or anti-angiogenic agents were 39% and 20%, respectively. The ORR difference among the regimens was not significant (p=0.43). With regard to PFS, naïve patients had higher PFS than treated patients (225d vs 103d, p=0.01) (**Figure 2B**). The treatment pattern was not significantly correlated with PFS (p=0.56) (**Figure 2C**).

### Predictive values of PD-L1 and TMB in response to ICI combination therapy

**Figure 3:**
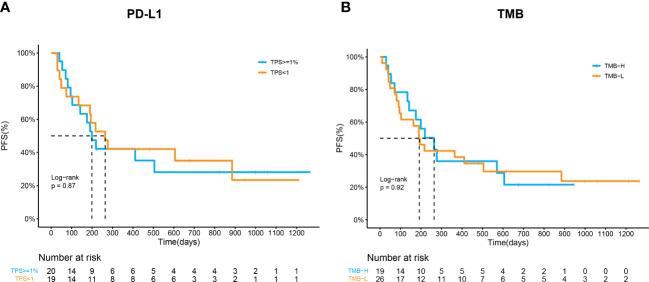
Predictive values of PD-L1 and TMB in response to ICI combination therapy. **(A)** Kaplan–Meier curves of PFS in patients by PD-L1 expression (p=0.87). **(B)** Kaplan–Meier curves of PFS in patients by TMB (p=0.92).

Considering that PD-L1 and TMB are the most widely used biomarkers in immunotherapy, their predictive roles in ICI combination therapy were also evaluated. Baseline tissue samples from 45 patients were collected for analysis. The expression of PD-L1 was measured by IHC in 39 patients. The ORR of patients with PD-L1 expression ≥1% or PD-L1 expression < 1% was not significantly different (52.6% vs. 40.0%, p=0.53). The mPFS of patients who had PD-L1 ≥1% was longer than that of those who did not, despite the trend was not significant (264d vs. 199d, p=0.87, **Figure 3A**). On the other hand, we spilt the cohort into the TMB-H (TMB>10 muts/Mb) or TMB-L (TMB ≤ 10 muts/Mb) group based on NGS results. The ORR and PFS of the two groups were similar (57.8% vs. 38.5%, p=0.24; 264d vs. 192d, p=0.92, **Figure 3B**), which implied a poor predictive value of TMB in ICI combination therapy.

### Gene mutations have potential to predict prognosis of NSCLC

**Figure 4:**
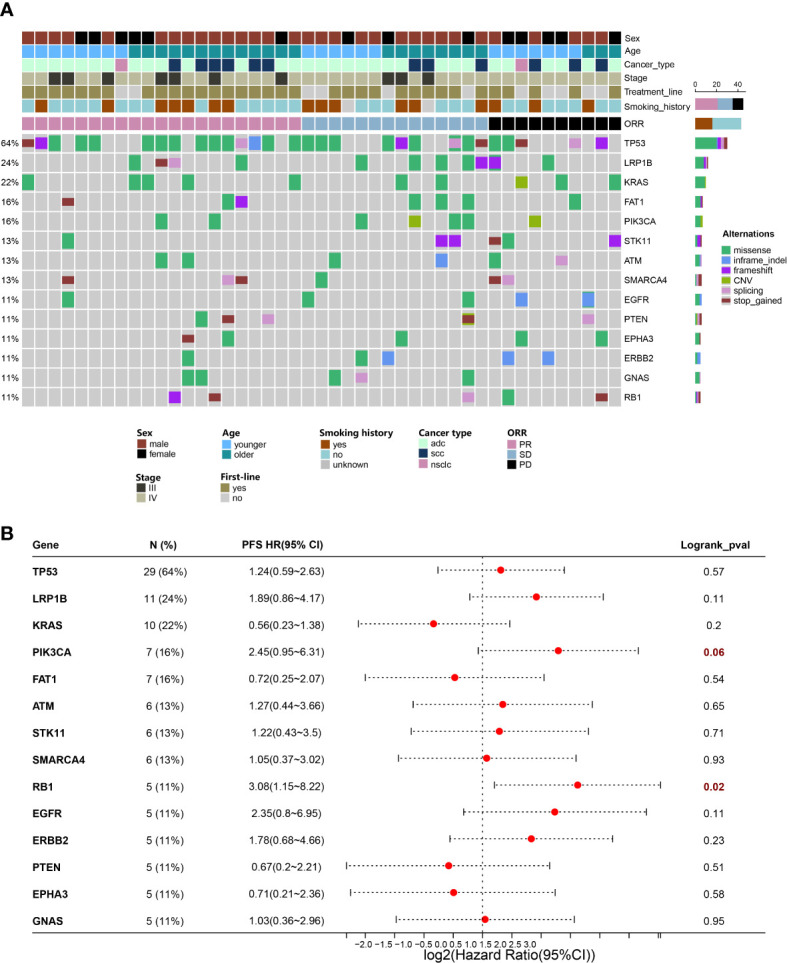
Gene mutations have potential to predict prognosis of NSCLC. **(A)** Distribution of genetic alterations in the study cohort. Onco-plot showing mutated genes of patients >=5 in the study cohort. Each column represents one patient, and clinical characteristics of each patient were indicated in the top panels. **(B)** Associations of genetic mutations with outcomes of ICI combination therapy. Forest plot presenting HRs of PFS comparing patients with and without gene mutations.

A 425-gene panel was used to assess the gene alterations in the specimens. The mutational profile was summarized in an onco-plot (**Figure 4A**). In this study, alterations detected in more than 5 patients were recorded to determine their relationship with ORR and PFS. TP53, LRP1B, and KRAS were ranked as the top three most frequently mutated genes, with frequencies of approximately 64%, 24%, and 22%, respectively. No gene alteration was significantly associated with ORR (**Table S2**). In contrast, specific gene mutations were significantly associated with PFS (**Figure 4B**). Univariate analysis revealed that alterations in RB1 and PIK3CA were risk factors. Their HRs were 3.08 (95%CI:1.15-8.22, p=0.02) and 2.45 (95%CI:0.95-6.31, p=0.06). Multivariate analysis was conducted (**Table 2**). RB1 mutations showing independent predictive values after multivariable adjustment to the line of treatment were selected as prognostic candidates (HR:2.78, 95%CI:1.03-7.50, p=0.04).

**Table 2:** Univariate and Multivariate Cox regression analyses of PFS.

Factor	Populations (%)	Univariable		Multivariable	
		HR (95% CI)	P value	HR (95% CI)	P value
First_line	38 (72%)	0.43 (0.22-0.83)	0.01	0.44 (0.20-0.95)	0.04
RB1	5 (11%)	3.08 (1.15-8.23)	0.02	2.78 (1.03-7.50)	0.04

### RB1 variations correlate with adverse outcomes of ICI combination therapy

**Figure 5:**
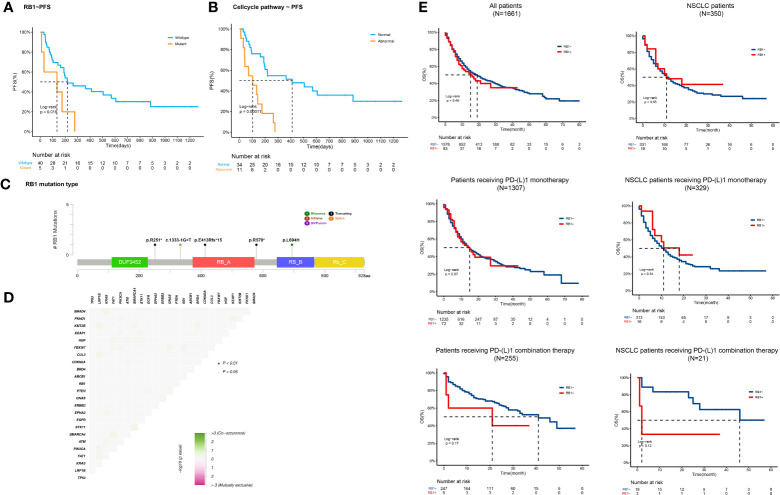
RB1 variations correlate with adverse outcomes of ICI combination therapy **(A, B)** Kaplan-Meier curves of PFS comparing patients with and without **(A)** *RB1* variations (134d vs 219d, p=0.018) or **(B)** cell-cycle pathway gene alterations (103d vs 411d, p<;0.001). **(C)** Lollipop plot showing detailed *RB1* mutations (p.R251*, c.1333-1G>T, p.E413Rfs*15, p.R579*, and p.L694H) detected in our cohort. **(D)** Correlation heatmap of gene alterations detected in our study. No co-mutation significantly associated with RB1 mutations was identified. **(E)** OS Kaplan-Meier curves of normal or abnormal RB1 patients/NSCLC patients (n=1661, n=350), patients/NSCLC patients receiving PD-(L)1 monotherapy (n=1307, n=329), and patients/NSCLC patients receiving PD-(L)1 combination therapy (n=255, n=21).

In this study, we focused on predicting the capacity of RB1 mutations. The mutation frequency of RB1 in our cohort was 11% (n=5). All five patients received ICI plus chemotherapy. Compared with patients with wild-type RB1, those with mutated RB1 had decreased mPFS (219d vs 134d, HR=3.08 (1.15-8.23), p=0.018) (**Figure 5A**). In the immune-chemo combination subgroup, RB1 mutation remained associated with poor outcome (PFS=264d vs 134d, HR=3.49 (1.20-10.18), p=0.01).Based on functional prediction tools, all detected RB1 mutations were inactivation mutations (**Figure 5C**). No other gene aberrations were significantly associated with the RB1 mutations (**Figure 5D**). However, it is worth noting that all the patients with RB1 mutations simultaneously presented with TP53 mutations. RB1 interacts with multiple cyclins and cyclin-dependent kinases (CDKs) to regulate the cell cycle pathway (23). Thus, we hypothesized that molecular alterations in cell cycle pathway-related genes might interfere with the therapeutic response to ICI combination regimens. Patients harboring at least one of the cell cycle pathway mutations were significantly associated with inferior PFS compared with those without these mutations (103d vs 411d, p<0.001) (**Figure 5B**). The HR for the abnormal cell cycle pathway was 4.27 (1.92-9.49). Multivariate analysis proved that the cell-cycle pathway mutation was significantly associated with poorer prognosis (p=0.001).We further analyzed prognostic data from TCGA included pan-cancer (24) and NSCLC (10). Results showed that the overall survival (OS) difference between patients with wild type RB1 or mutated RB1 was not significant. For patients receiving PD-(L)1 monotherapy, RB1 status also had no effects on OS. However, compared with normal patients receiving PD-(L)1 combination therapy, RB1 mutated patients had relatively poorer prognosis and the difference was more notable among NSCLC patients (**Figure 5E**). Although the significance was limited by the sample size, these results also suggested that RB1 variations were associated with outcomes of ICI combination therapy.

### CIN associated with abnormal cell cycle pathway indicates poor prognosis

**Figure 6:**
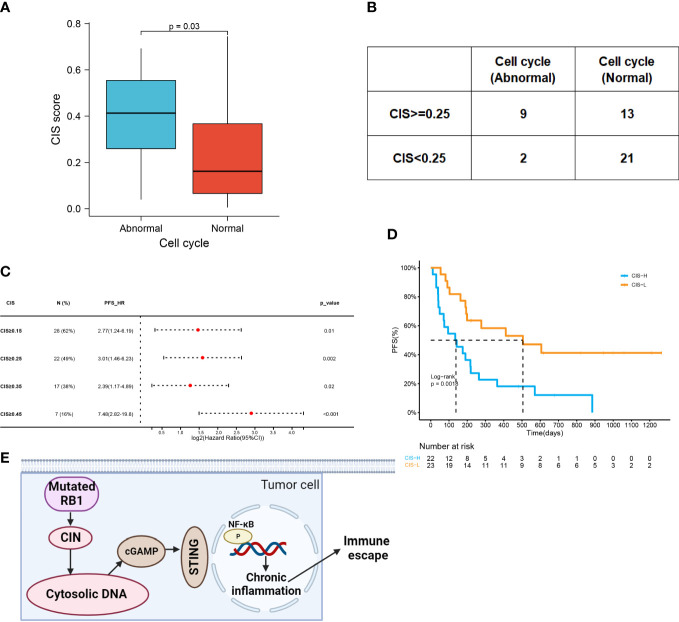
CIN associated with abnormal cell cycle pathway indicates poor prognosis. **(A)** CIS in the abnormal cell cycle group and the normal cell cycle group (0.41 vs 0.17, p=0.03). **(B)** Comparison of CIS level in patients with the abnormal or normal cell cycle (p=0.02). The cutoff point of CIS was 0.25. **(C)** Forest plot of HRs for PFS comparing patients at varying cutoff points of CIS. **(D)** Kaplan–Meier curves of PFS in patients with CIS ≥0.25 or with CIS<;0.25 (138d vs 505d, HR=3.01 (1.46-6.23), p=0.0018). **(E)** Aggravated CIN induced by RB1 mutations could promote the generation of cytosolic DNA, which triggered immune escape by regulating cGAS-STING signaling and NF-κB activation to cause chronic inflammation. Figure created using BioRender.com.

Genetic instability may disrupt chromosome stability. CIN has been widely observed in multiple malignancies. The chromosomal instability score (CIS) was used to describe the extent of CIN. Based on the finding that the cell cycle pathway is associated with CIN (25), we analyzed the effects of cell cycle pathway mutations on CIS. The results showed that patients with abnormal cell cycle pathways had higher CIS (0.41 vs 0.17, p=0.03), suggesting that the mutated cell cycle pathway might induce an unstable genome (**Figure 6A**). We settled 0.25 as the cutoff point of CIS and confirmed the significant association between CIS and cell cycle pathway status (p=0.02) (**Figure 6B**). These results showed that patients who encountered more aberrations in the cell cycle pathway had higher CIS.

A total of 28 patients presented with CIS ≥0.15, the HR of which was 2.77 (95% CI:1.24-6.19). Increasing the cutoff points of CIS increased HR (**Figure 6C**). By elevating the threshold to 0.25 or 0.45, the HR of CIS was 3.01 (95% CI:1.46-6.23) or 7.48 (95% CI:2.82-19.8). However, HR of CIS ≥0.35 was only 2.39 (95% CI: 1.17-4.89). According to a CIS cutoff of 0.25, we compared the PFS of the high- and low-CIS groups (**Figure 6D**). Low CIS was a strong indicator of favorable PFS among patients (505d vs 138d, p=0.0018). Taken together, the mutated cell cycle pathway following CIN might inhibit the response to ICI combined therapy (**Figure 6E**).

### ctDNA status as a potential biomarker for ICI combined therapy

**Figure 7:**
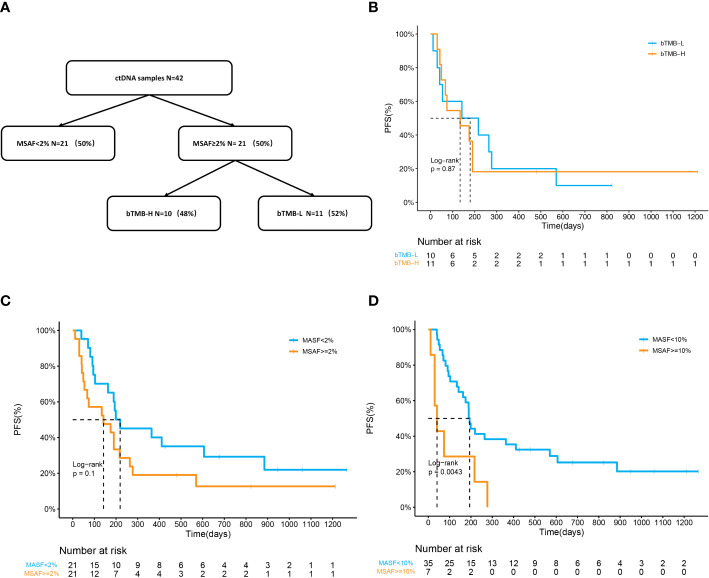
ctDNA status as a potential biomarker for ICI combined therapy. **(A)** Flow diagram of plasma samples stratified by MSAF and bTMB. **(B)** Kaplan–Meier curves of PFS in patients divided by bTMB. The mPFS was 179 days in the bTMB-H group and 134 days in the bTMB-L group (p=0.87). **(C, D)** Kaplan–Meier curves of PFS in patients divided by MSAF. **(C)** mPFS was 219d in the MSAF<;2% group and 142d in the MSAF≥2% group (p=0.1). HR of MSAF≥2% for PFS is 1.77 (95%CI:0.89-3.55). **(D)** mPFS was 194d in the MSAF<;10% group and 41d in the MSAF≥10% group (p=0.0043). HR of MSAF≥10% for PFS is 3.28 (95%CI:1.39-7.77).

42 baseline blood samples were available for NGS including MSAF and bTMB calculations. MSAF indicates the release of ctDNA with a frequently used cutoff of 2% (26). Patients were divided into two groups (MSAF < 2%, n=21; MSAF≥2%, n=21). Patients with MSAF ≥2% that met the minimum amount of ctDNA were further allocated to the bTMB-H or bTMB-L subgroups (bTMB≥10 muts/Mb, n=11; bTMB < 10 muts/Mb, n=10) (27) (**Figure 7A**). No significant correlation between bTMB and prognosis was identified, as the mPFS of the bTMB-H and bTMB-L group was 179d and 134d (p=0.80) (**Figure 7B**). The mPFS of patients with MSAF≥2% was 142d, while that of patients with MSAF<2% was 219d (p=0.1) (**Figure 7C**). HR of MSAF≥2% was 1.77 (95%CI:0.89-3.55). mPFS was significantly shorter in patients with MSAF≥10% than in those with MSAF<10% (41d vs 194d, HR:3.28(1.39-7.77), p=0.0043, **Figure 7D**). We examined the association between MSAF and clinical variables and found no significant association (**Table S3**). Consequently, NSCLC patients with a lower MSAF might respond better to ICI combination therapy.

## Discussion

NSCLC is the most common lung cancer with a high mortality rate, metastasis, recurrence, and multidrug resistance (28). Although ICIs have become a promising therapeutic modality for NSCLC, maximizing the response to first-line treatment is critical. Chemotherapy and anti-angiogenic agents can augment antitumor immunity by impeding the immunosuppressive tumor microenvironment (29). Clinical trials including KEYNOTE-189, IMpower150, and IMpower130 have demonstrated improved OS with ICI combined chemotherapy (30, 31). However, predictive biomarkers for the benefits of immunotherapy might not be optimal for ICI combined treatment. For instance, atezolizumab combined with chemotherapy and bevacizumab has been approved as a first-line treatment for NSCLC irrespective of PD-L1 expression (32). Meanwhile, there is a greater likelihood for a high TMB to reflect acquired resistance rather than immune benefits (33). These phenomena could be partially explained by the increase in immunogenicity induced by cytotoxic approaches.

Considering that NSCLC harbors high rates of somatic mutations and genomic rearrangements (34), we conducted a single-arm study consisting of 53 NSCLC patients receiving ICI combination therapy and employed NGS to determine key progression-related biomarkers. The ORR of all patients was 39.6%, which is similar to that in previous reports (33%-47%) (35). mPFS in our cohort was 190d. The line of treatment was the only clinical feature that significantly associated with the response and prognosis. Patients in the first-line treatment had significantly better ORR and PFS than those in the second-line or later-line treatment. Neither PD-L1 nor TMB exhibited high prognostic value in this study, suggesting the limitation of classical biomarkers in ICI combination therapy.

The genomic landscape of patients was determined using NGS. None of the gene mutations was significantly associated with ORR. By contrast, specific composite somatic mutations have the potential to serve as PFS-related biomarkers. Univariate analysis indicated that RB1 and PIK3CA mutations were deleterious factors for PFS. Further multivariable analysis verified that the RB1 mutation was harmful to the prognosis of combined treatment. Consistent with our results, bioinformatic analysis showed that RB1 mutation was especially deleterious to NSCLC patients receiving PD-(L)1 combination therapy. RB1 is a tumor suppressor gene that is mutated in various human cancers (23). pRB, translated by RB1, is a chromatin-associated protein that limits the transcription of cell cycle genes by suppressing E2F (36, 37). The mutation frequency of RB1 in our cohort was 11%, which was slightly higher than that reported in previous studies (8.2%) (38). All RB1 mutations were inactivated in this study. RB1 mutations have been observed in several solid tumors and are associated with poor outcomes in early stage and advanced NSCLC (39). RB1 is a prognostic candidate for immunotherapy, as decreased expression of RB1 in hepatoma or bladder cancer has been correlated with a lack of immune response (40, 41). It has also been reported that six NSCLC patients with RB1 mutations failed to respond to immunotherapy (38). RB1 mutations in our study decreased the efficacy of ICI combination therapy in terms of PFS, thereby highlighting their prognostic value. Intriguingly, co-mutation analysis showed that no other gene alterations were significantly correlated with RB1 mutations. However, all RB1alterations in our study co-occurred with TP53 mutations, which is in accordance with previous reports, suggesting that p53 signaling is involved in the control of the cell cycle (22).

pRB is a master regulator of the cell cycle and its inactivation elongates the cell cycle (23). The cell cycle pathway, consisting of CDKs, CDK inhibitors, and cyclins, is frequently altered across many different tumor types (22). Similar to mutated RB1, abnormal cell cycle pathway is closely related to decreased PFS in ICI combination therapy. Both RB1 defects and cell cycle deregulation could further contribute to CIN (25, 42). In the present study, abnormal cell cycle pathways were significantly associated with a high incidence of CIN. Increased CIN indicates a worse response to ICI combination therapy. CIN is part of genomic instability and is characterized by copy number alterations in whole or parts of chromosomes, participating in the initiation and progression of tumors (43, 44). Tumor suppressors such as p53 and pRB protect against CIN (45). In NSCLC, high CIN expression exerts oncogenic functions and reflects a poor prognosis (46, 47). In our study, the optimal cut-off point for CIS was determined as 0.25 in our study. Consistent with the above reports, high CIS was significantly associated with an unfavorable prognosis of ICI combination therapy. The rationale behind this phenomenon is likely associated with cytosolic DNA. Errors in chromosome segregation create a preponderance of micronuclei, whose rupture spills genomic DNA into the cytosol (48). Cytosolic DNA can facilitate an immunosuppressive microenvironment and trigger immune evasion by regulating cGAS-STING signaling (49). Collectively, RB1 mutations may induce resistance to ICI combined therapy by disrupting cell cycle control and promoting the formation of chromosomal aberrations. Aggravated CIN may trigger immune suppression *via* chronic inflammation (**Figure 3E**).

Moreover, the plasma tumor fraction tested by NGS is important for predicting the prognosis. The value of bTMB in differentiating OS benefits is limited (27). In contrast, MSAF, which represents tumor purity, is closely associated with prognosis. It was established that patients with higher MSAF had worse OS than those with lower MSAF in the POPLAR and OAK cohorts (16). In our study, MSAF≥10% was a significant negative prognostic factor for PFS. Dynamic detection of MSAF can track responses to ICI in NSCLC (50). Therefore, MSAF representing ctDNA fractions can provide independent information in the context of established clinical biomarkers.

Our study has several limitations. It’s worth noting that the immune combination therapy comprises both chemotherapy and anti-angiogenic therapies. Further large-scale studies are necessary to fully confirm the predictive value of RB1 and its related cell-cycle pathway gene alterations, and with respect to different treatment scheme. Another limitation is the absence of *in vitro* experiments to elucidate the underlying mechanisms of RB1 mutations. Additionally, we did not collect sufficient samples upon disease progression for further drug resistance studies. Although limited by a moderate sample size, our study identified RB1 as a strong negative predictor of poor outcome to anti-PD-(L)1 combination therapy both in our study cohort and in the external validation cohort. Future studies should focus on expanding the cohort size and include functional analysis to elucidate the underlying mechanisms of disease progression.

In conclusion, this pilot study supports further research on RB1 for predicting the efficacy of ICI combination therapy in NSCLC. RB1 aberrations may attenuate the response in concert with abnormal signaling pathways and an unstable chromosome. Determining RB1 status may serve as prognostic indicators to complement traditional biomarkers and guide clinical treatment.

## Data availability statement

The data presented in the study are deposited in the Genome Sequence Archive (Genomics, Proteomics & Bioinformatics 2021) in National Genomics Data Center (Nucleic Acids Res 2022), China National Center for Bioinformation/Beijing Institute of Genomics, Chinese Academy of Sciences (GSA-Human), publicly accessible at https://ngdc.cncb.ac.cn/gsa-human, accession number HRA004817.

## Ethics statement

The studies involving human participants were reviewed and approved by Institutional Review Board of Jiangsu Province Hospital. The patients/participants provided their written informed consent to participate in this study. Written informed consent was obtained from the individual(s) for the publication of any potentially identifiable images or data included in this article.

All the tissue samples involved in this study came from previous paraffin-embedded surgical specimens derived from patients with NSCLC, informed consent has been signed and received. The study protocol was approved by the Institutional Review Board of Jiangsu Province Hospital (2022-SR-042). with the Ethics statement.

## Author contributions

K-HL conceived the study. QW and TY drafted the manuscript and designed figures. F-FW and J-NY refined the experimental design and implemented the methodology used in this study. YS and Z-HK performed data analysis. All authors contributed to the article and approved the submitted version.

## References

[1] Reck M Rodriguez-Abreu D Robinson AG Hui R Csoszi T Fulop A. Pembrolizumab versus chemotherapy for PD-L1-Positive non-Small-Cell lung cancer. N Engl J Med (2016) 375:1823–33. doi: 10.1056/NEJMoa1606774 27718847

[2] Judd J Borghaei H. Combining immunotherapy and chemotherapy for non-small cell lung cancer. Thorac Surg Clinics (2020) 30:199–206. doi: 10.1016/j.thorsurg.2020.01.006 32327178

[3] Vafaei S Zekiy AO Khanamir RA Zaman BA Ghayourvahdat A Azimizonuzi H. Combination therapy with immune checkpoint inhibitors (ICIs); a new frontier. Cancer Cell Int (2022) 22:2. doi: 10.1186/s12935-021-02407-8 34980128 PMC8725311

[4] Cortes J Cescon DW Rugo HS Nowecki Z Im SA Yusof MM. Pembrolizumab plus chemotherapy versus placebo plus chemotherapy for previously untreated locally recurrent inoperable or metastatic triple-negative breast cancer (KEYNOTE-355): a randomised, placebo-controlled, double-blind, phase 3 clinical trial. Lancet (2020) 396:1817–28. doi: 10.1016/S0140-6736(20)32531-9 33278935

[5] Bylicki O Barazzutti H Paleiron N Margery J Assie JB Chouaid C. First-line treatment of non-Small-Cell lung cancer (NSCLC) with immune checkpoint inhibitors. BioDrugs: Clin immunotherapeutics biopharmaceuticals Gene Ther (2019) 33:159–71. doi: 10.1007/s40259-019-00339-4 30825132

[6] Burgess EF Livasy C Hartman A Robinson MM Symanowski J Naso C. Discordance of high PD-L1 expression in primary and metastatic urothelial carcinoma lesions. Urologic Oncol (2019) 37(5):299.e219–299 .e225. doi: 10.1016/j.urolonc.2019.01.002 30660491

[7] Rizvi NA Hellmann MD Snyder A Kvistborg P Makarov V Havel JJ. Cancer immunology. mutational landscape determines sensitivity to PD-1 blockade in non-small cell lung cancer. Science (2015) 348:124–8. doi: 10.1126/science.aaa1348 PMC4993154 25765070

[8] McGrail DJ Pilie PG Rashid NU Voorwerk L Slagter M Kok M. High tumor mutation burden fails to predict immune checkpoint blockade response across all cancer types. Ann Oncol (2021) 32:661–72. doi: 10.1016/j.annonc.2021.02.006 PMC8053682 33736924

[9] Kim SY Halmos B. Choosing the best first-line therapy: NSCLC with no actionable oncogenic driver. Lung Cancer Manage (2020) 9:LMT36. doi: 10.2217/lmt-2020-0003 PMC7399613 32774467

[10] Rizvi H Sanchez-Vega F La K Chatila W Jonsson P Halpenny D. Molecular determinants of response to anti-programmed cell death (PD)-1 and anti-programmed death-ligand 1 (PD-L1) blockade in patients with non-Small-Cell lung cancer profiled with targeted next-generation sequencing. J Clin Oncol (2018) 36:633–41. doi: 10.1200/JCO.2017.75.3384 PMC6075848 29337640

[11] Li L Li M Wang X. Cancer type-dependent correlations between TP53 mutations and antitumor immunity. DNA Repair (2020) 88:102785. doi: 10.1016/j.dnarep.2020.102785 32007736

[12] Camidge DR Doebele RC Kerr KM. Comparing and contrasting predictive biomarkers for immunotherapy and targeted therapy of NSCLC. Nat Rev Clin Oncol (2019) 16:341–55. doi: 10.1038/s41571-019-0173-9 30718843

[13] Pan YH Zhang JX Chen X Liu F Cao JZ Chen Y. Predictive value of the TP53/PIK3CA/ATM mutation classifier for patients with bladder cancer responding to immune checkpoint inhibitor therapy. Front Immunol (2021) 12:643282. doi: 10.3389/fimmu.2021.643282 34421886 PMC8371040

[14] Kato S Goodman A Walavalkar V Barkauskas DA Sharabi A Kurzrock R. Hyperprogressors after immunotherapy: analysis of genomic alterations associated with accelerated growth rate. Clin Cancer Res (2017) 23:4242–50. doi: 10.1158/1078-0432.CCR-16-3133 PMC5647162 28351930

[15] Pietrasz D Pecuchet N Garlan F Didelot A Dubreuil O Doat S. Plasma circulating tumor DNA in pancreatic cancer patients is a prognostic marker. Clin Cancer Res (2017) 23:116–23. doi: 10.1158/1078-0432.CCR-16-0806 27993964

[16] Wang Z Duan J Wang G Zhao J Xu J Han J. Allele frequency-adjusted blood-based tumor mutational burden as a predictor of overall survival for patients with NSCLC treated with PD-(L)1 inhibitors. J Thorac Oncol (2020) 15:556–67. doi: 10.1016/j.jtho.2019.12.001 31843683

[17] Chen YT Seeruttun SR Wu XY Wang ZX. Maximum somatic allele frequency in combination with blood-based tumor mutational burden to predict the efficacy of atezolizumab in advanced non-small cell lung cancer: a pooled analysis of the randomized POPLAR and OAK studies. Front Oncol (2019) 9. doi: 10.3389/fonc.2019.01432 PMC6929100 31921683

[18] Wang K Li M Hakonarson H. ANNOVAR: functional annotation of genetic variants from high-throughput sequencing data. Nucleic Acids Res (2010) 38:e164. doi: 10.1093/nar/gkq603 20601685 PMC2938201

[19] Ng PC Henikoff S. SIFT: predicting amino acid changes that affect protein function. Nucleic Acids Res (2003) 31:3812–4. doi: 10.1093/nar/gkg509 PMC168916 12824425

[20] Adzhubei I Jordan DM Sunyaev SR. Predicting functional effect of human missense mutations using PolyPhen-2. Curr Protoc Hum Genet (2013) Chapter 7:Unit7 20. doi: 10.1002/0471142905.hg0720s76 PMC4480630 23315928

[21] Ying J Yang L Yin JC Xia G Xing M Chen X. Additive effects of variants of unknown significance in replication repair-associated DNA polymerase genes on mutational burden and prognosis across diverse cancers. J Immunother Cancer (2021) 9:e002336. doi: 10.1136/jitc-2021-002336 34479923 PMC8420654

[22] Sanchez-Vega F Mina M Armenia J Chatila WK Luna A La KC. Oncogenic signaling pathways in the cancer genome atlas. Cell (2018) 173:321–337.e310. doi: 10.1016/j.cell.2018.03.035 29625050 PMC6070353

[23] Dyson NJ. RB1: a prototype tumor suppressor and an enigma. Genes Dev (2016) 30:1492–502. doi: 10.1101/gad.282145.116 PMC4949322 27401552

[24] Samstein RM Lee CH Shoushtari AN Hellmann MD Shen R Janjigian YY. Tumor mutational load predicts survival after immunotherapy across multiple cancer types. Nat Genet (2019) 51:202–6. doi: 10.1038/s41588-018-0312-8 PMC6365097 30643254

[25] Malumbres M Barbacid M. Cell cycle, CDKs and cancer: a changing paradigm. Nat Rev Cancer (2009) 9:153–66. doi: 10.1038/nrc2602 19238148

[26] Fang W Ma Y Yin JC Zhou H Wang F Bao H. Combinatorial assessment of ctDNA release and mutational burden predicts anti-PD(L)1 therapy outcome in nonsmall-cell lung cancer. Clin Trans Med (2020) 10:331–6. doi: 10.1002/ctm2.8 PMC7240844 32508057

[27] Gandara DR Paul SM Kowanetz M Schleifman E Zou W Li Y. Blood-based tumor mutational burden as a predictor of clinical benefit in non-small-cell lung cancer patients treated with atezolizumab. Nat Med (2018) 24:1441–8. doi: 10.1038/s41591-018-0134-3 30082870

[28] Siegel RL Miller KD Jemal A. Cancer statistics, 2020. CA Cancer J Clin (2020) 70:7–30. doi: 10.3322/caac.21590 31912902

[29] Patel SA Minn AJ. Combination cancer therapy with immune checkpoint blockade: mechanisms and strategies. Immunity (2018) 48:417–33. doi: 10.1016/j.immuni.2018.03.007 PMC6948191 29562193

[30] Reck M Rodriguez-Abreu D Robinson AG Hui R Csoszi T Fulop A. Updated analysis of KEYNOTE-024: pembrolizumab versus platinum-based chemotherapy for advanced non-Small-Cell lung cancer with PD-L1 tumor proportion score of 50% or greater. J Clin Oncol (2019) 37:537–46. doi: 10.1200/JCO.18.00149 30620668

[31] Gandhi L Rodriguez-Abreu D Gadgeel S Esteban E Felip E De Angelis F. Pembrolizumab plus chemotherapy in metastatic non-Small-Cell lung cancer. N Engl J Med (2018) 378:2078–92. doi: 10.1056/NEJMoa1801005 29658856

[32] Socinski MA Nishio M Jotte RM Cappuzzo F Orlandi F Stroyakovskiy D. IMpower150 final overall survival analyses for atezolizumab plus bevacizumab and chemotherapy in first-line metastatic nonsquamous NSCLC. J Thorac Oncol (2021) 16:1909–24. doi: 10.1016/j.jtho.2021.07.009 34311108

[33] McGranahan N Furness AJ Rosenthal R Ramskov S Lyngaa R Saini SK. Clonal neoantigens elicit T cell immunoreactivity and sensitivity to immune checkpoint blockade. Science (2016) 351:1463–9. doi: 10.1126/science.aaf1490 PMC4984254 26940869

[34] Cancer Genome Atlas Research N. Comprehensive molecular profiling of lung adenocarcinoma. Nature (2014) 511:543–50. doi: 10.1038/nature13385 PMC4231481 25079552

[35] Rizvi NA Hellmann MD Brahmer JR Juergens RA Borghaei H Gettinger S. Nivolumab in combination with platinum-based doublet chemotherapy for first-line treatment of advanced non-Small-Cell lung cancer. J Clin Oncol (2016) 34:2969–79. doi: 10.1200/JCO.2016.66.9861 PMC5569693 27354481

[36] Hernando E Nahle Z Juan G Diaz-Rodriguez E Alaminos M Hemann M. Rb Inactivation promotes genomic instability by uncoupling cell cycle progression from mitotic control. Nature (2004) 430:797–802. doi: 10.1038/nature02820 15306814

[37] Calo E Quintero-Estades JA Danielian PS Nedelcu S Berman SD Lees JA. Rb Regulates fate choice and lineage commitment. Vivo Nat (2010) 466:1110–4. doi: 10.1038/nature09264 PMC2933655 20686481

[38] Bhateja P Chiu M Wildey G Lipka MB Fu P Yang MCL. Retinoblastoma mutation predicts poor outcomes in advanced non small cell lung cancer. Cancer Med (2019) 8:1459–66. doi: 10.1002/cam4.2023 PMC6488103 30773851

[39] Choi S Kim HR Sung CO Kim J Kim S Ahn SM. Genomic alterations in the RB pathway indicate prognostic outcomes of early-stage lung adenocarcinoma. Clin Cancer Res (2015) 21:2613–23. doi: 10.1158/1078-0432.CCR-14-0519 25294902

[40] Hutcheson J Bourgo RJ Balaji U Ertel A Witkiewicz AK Knudsen ES. Retinoblastoma protein potentiates the innate immune response in hepatocytes: significance for hepatocellular carcinoma. Hepatology (2014) 60:1231–40. doi: 10.1002/hep.27217 PMC4482134 24824777

[41] Cormio L Tolve I Annese P Saracino A Zamparese R Sanguedolce F. Retinoblastoma protein expression predicts response to bacillus calmette-guerin immunotherapy in patients with T1G3 bladder cancer. Urologic Oncol (2010) 28:285–9. doi: 10.1016/j.urolonc.2008.08.003 18976938

[42] Nath S Chowdhury A Dey S Roychoudhury A Ganguly A Bhattacharyya D. Deregulation of Rb-E2F1 axis causes chromosomal instability by engaging the transactivation function of Cdc20-anaphase-promoting complex/cyclosome. Mol Cell Biol (2015) 35:356–69. doi: 10.1128/MCB.00868-14 PMC4272426 25368385

[43] Negrini S Gorgoulis VG Halazonetis TD. Genomic instability–an evolving hallmark of cancer. Nat Rev Mol Cell Biol (2010) 11:220–8. doi: 10.1038/nrm2858 20177397

[44] Bakhoum SF Landau DA. Chromosomal instability as a driver of tumor heterogeneity and evolution. Cold Spring Harb Perspect Med (2017) 7(6):a029611. doi: 10.1101/cshperspect.a029611 28213433 PMC5453382

[45] Voutsadakis IA. Clinical implications of chromosomal instability (CIN) and kinetochore abnormalities in breast cancers. Mol Diagn Ther (2019) 23:707–21. doi: 10.1007/s40291-019-00420-2 31372940

[46] Monteverde T Sahoo S La Montagna M Magee P Shi L Lee D. CKAP2L promotes non-small cell lung cancer progression through regulation of transcription elongation. Cancer Res (2021) 81:1719–31. doi: 10.1158/0008-5472.CAN-20-1968 33472893

[47] Nakamura H Saji H Idiris A Kawasaki N Hosaka M Ogata A. Chromosomal instability detected by fluorescence *in situ* hybridization in surgical specimens of non-small cell lung cancer is associated with poor survival. Clin Cancer Res (2003) 9:2294–9. doi: 10.1016/S0169-5002(03)92602-X 12796398

[48] Bakhoum SF Ngo B Laughney AM Cavallo JA Murphy CJ Ly P. Chromosomal instability drives metastasis through a cytosolic DNA response. Nature (2018) 553:467–72. doi: 10.1038/nature25432 PMC5785464 29342134

[49] Bakhoum SF Cantley LC. The multifaceted role of chromosomal instability in cancer and its microenvironment. Cell (2018) 174:1347–60. doi: 10.1016/j.cell.2018.08.027 PMC6136429 30193109

[50] Li L Wang Y Shi W Zhu M Liu Z Luo N. Serial ultra-deep sequencing of circulating tumor DNA reveals the clonal evolution in non-small cell lung cancer patients treated with anti-PD1 immunotherapy. Cancer Med (2019) 8:7669–78. doi: 10.1002/cam4.2632 PMC6912064 31692284

