# Supplementary material for: RB1 aberrations predict outcomes of immune checkpoint inhibitor combination therapy in NSCLC
Source: Front Oncol. 2023 Jun 27;13:1172728. doi: 10.3389/fonc.2023.1172728 (PMC10334286; doi:10.3389/fonc.2023.1172728)
Supplement: Supplementary file 1 [file Table_1.docx]

Supplement

Table S1

Characteristics of the patients with different response to ICI combination therapy.

| **Clinical information** | **SD+PD** | **PR** | **P value** |
| --- | --- | --- | --- |
| **Age** |  |  | **0.51** |
| Median [min-max] | 67 [41,79] | 70 [54,80] |  |
| **Sex** |  |  |  |
| female | 11(65%) | 6(35%) | **0.77** |
| male | 21(58%) | 15(42%) |  |
| **Smoking history** |  |  |  |
| no | 13(65%) | 7(35%) | **0.57** |
| yes | 17(55%) | 14(45%) |  |
| NA | 2(100%) | 0(0%) |  |
| **Cancer type** |  |  |  |
| adenocarcinoma (adc) | 23(62%) | 14(38%) | **0.76** |
| squamous cell cancer (scc) | 8(57%) | 6(43%) |  |
| others | 1(50%) | 1(50%) |  |
| **Stage** |  |  |  |
| III | 4(36%) | 7(64%) | **0.09** |
| IV | 28(67%) | 14(33%) |  |
| **First-line** |  |  |  |
| yes | 19(50%) | 19(50%) | **0.03** |
| no | 13(87%) | 2(13%) |  |
| **Treatment** |  |  |  |
| Anti-PD-(L)1 and Anti-angiogenic | 4(80%) | 1(20%) | **0.43** |
| Anti-PD-(L)1 and Chemo | 19(61%) | 12(39%) |  |
| Anti-PD-(L)1, Anti-angiogenic and Chemo | 7(47%) | 8(53%) |  |
| Others* | 2(100%) | 0(0%) |  |

*Anti-PD-(L)1 and Targeted therapy N=1; Anti-PD-(L)1, Anti-angiogenic and Targeted therapy N=1

Table S2

Comparison of ORR in patients with or without specific gene alterations.

| **Gene** | **N (%)** | **Mutant.ORR** | **Wildtype.ORR** | **Odds_ratio** | **p.result** |
| --- | --- | --- | --- | --- | --- |
| **TP53** | **29 (64%)** | **51.72%** | **37.50%** | **1.76** | **0.53** |
| **LRP1B** | **11 (24%)** | **36.36%** | **50.00%** | **0.58** | **0.50** |
| **KRAS** | **10 (22%)** | **50.00%** | **45.71%** | **1.18** | **>0.99** |
| **FAT1** | **7 (16%)** | **42.86%** | **47.37%** | **0.84** | **>0.99** |
| **PIK3CA** | **7 (16%)** | **28.57%** | **50.00%** | **0.41** | **0.42** |
| **ATM** | **6 (13%)** | **33.33%** | **48.72%** | **0.53** | **0.67** |
| **SMARCA4** | **6 (13%)** | **50.00%** | **46.15%** | **1.16** | **>0.99** |
| **STK11** | **6 (13%)** | **16.67%** | **51.28%** | **0.2** | **0.19** |
| **EGFR** | **5 (11%)** | **20.00%** | **50.00%** | **0.26** | **0.35** |
| **EPHA3** | **5 (11%)** | **40.00%** | **47.50%** | **0.74** | **>0.99** |
| **ERBB2** | **5 (11%)** | **20.00%** | **50.00%** | **0.26** | **0.35** |
| **GNAS** | **5 (11%)** | **40.00%** | **47.50%** | **0.74** | **>0.99** |
| **PTEN** | **5 (11%)** | **60.00%** | **45.00%** | **1.81** | **0.65** |
| **RB1** | **5 (11%)** | **40.00%** | **47.50%** | **0.74** | **>0.99** |

Table S3

Characteristics of patients with MSAF≥10% or＜10%. No significant correlation with MSAF was confirmed.

| **Characteristics** | **MSAF<10% (N=35)** | **MSAF≥10% (N=7)** | **P value** |  |
| --- | --- | --- | --- | --- |
| **Sex** |  |  | **>0.99** |  |
| **female** | **11(35%)** | **2(29%)** |  |  |
| **male** | **24(65%)** | **5(71%)** |  |  |
| **Age** |  |  | **0.61** |  |
| **median[min-max]** | **69.00[46,80]** | **63.00[52,76]** |  |  |
| **Smoking history** |  |  | **0.39** |  |
| **yes** | **11(31%)** | **4(57%)** |  |  |
| **no** | **22(63%)** | **3(43%)** |  |  |
| **NA** | **2(6%)** | **0(0%)** |  |  |
| **Cancer type** |  |  | **0.33** |  |
| **adc** | **26(74%)** | **3(43%)** |  |  |
| **scc** | **9(26%)** | **3(43%)** |  |  |
| **others** | **0(0%)** | **1(14%)** |  |  |
| **Stage** |  |  | **>0.99** |  |
| **III** | **5(14%)** | **1(14%)** |  |  |
| **IV** | **30(86%)** | **6(86%)** |  |  |
| **First-line** |  |  | **0.06** |  |
| **Yes** | **28(80%)** | **3(43%)** |  |  |
| **No** | **7(20%)** | **4(57%)** |  |  |
| **Treatment** |  |  | **0.63** |  |
| **Anti-PD-(L)1 and Anti-angiogenic** | **3(9%)** | **0(0%)** |  |  |
| **Anti-PD-(L)1 and Chemo** | **19(54%)** | **5(72%)** |  |  |
| **Anti-PD-(L)1, Anti-angiogenic and chemo** | **12(34%)** | **1(14%)** |  |  |
| **Others*** | **1(3%)** | **1(14%)** |  |  |
| * Anti-PD-(L)1 and Targeted therapy N =1; Anti-PD(L)1, Anti-angiogenic and Targeted therapy n=1. | | | |  |
|  |  |  |  |  |
