# Supplementary material for: RB1 aberrations predict outcomes of immune checkpoint inhibitor combination therapy in NSCLC
Source: Front Oncol. 2023 Jun 27;13:1172728. doi: 10.3389/fonc.2023.1172728 (PMC10334286; doi:10.3389/fonc.2023.1172728)
Supplement: Supplementary file 4 [file Table_4.docx]

### survival plot ###

setwd("")

library('openxlsx')

library('ggplot2')

library('stringr')

library("survival")

library("survminer")

mydat<-read.xlsx("",sheet=1,colNames = T)

fit <- survfit(Surv(PFS, PFS_status) ~ group, data = mydat,na.action=na.exclude)

survp1<-ggsurvplot(fit,xlab = "Time(days)", ylab = "PFS(%)",title="", legend.title = c(""), #legend=c(0.85,0.8),

break.x.by = 5, #x-axis interval

break.y.by = 0.2,

surv.scale = c("percent"),

#legend.labs = c("bTMB-L","bTMB-H"),###ʵ????0????????1

pval=TRUE,pval.size = 4,pval.coord=c(0.8,0.2),pval.method=TRUE,pval.method.coord=c(0.2,0.25),#add p-value

conf.int = F, #confidence interval

axes.offset=TRUE, #plot axes start at origin

censor.shape = 124,censor.size = 2,

#linetype = "strata", # Change line type by groups

#linetype = c("TP53"),

surv.median.line = "hv", # Specify median survival

#ggtheme = theme_bw(), # Change ggplot2 theme

#palette = c("#00aeef","#f7931d","#00aeef","#f7931d"),

color=c("blue"),

palette = "lancet",

risk.table = TRUE, risk.table.y.text.col=TRUE,tables.height = 0.15,table.axes.offset=FALSE,tables.theme = clean_theme()

)

survp1

fit <- survfit(Surv(PFS, PFS_status) ~ group, data = mydat,na.action=na.exclude)

summary(fit)$table

#----------------------------------------------------------------------------------------------

### gene analysis related to PFS ###

mydat<-read.xlsx("",sheet=1,colNames = T)

covariates <- colnames(mydat)[6:ncol(mydat)]

mult_formula_f <- sapply(covariates,function(x)as.formula(paste("Surv(PFS,PFS_status)~", x)))

mult_model_f <- lapply(mult_formula_f, function(x){coxph(x, data=mydat)})

mult_result_f <- lapply(mult_model_f,

function(x){

x <- summary(x)

co <- signif(x$coefficients[, "coef"][1], digits = 5)

hr <- round(x$coefficients[, "exp(coef)"][1], digits = 2)

p.waldz <- signif(x$coefficients[, "Pr(>|z|)"], digits = 3)

p.logrank <- signif(x$sctest["pvalue"], digits = 3)

HR.confint.lower <- round(x$conf.int[,"lower .95"][1], digits =2)

HR.confint.upper <- round(x$conf.int[,"upper .95"][1], digits =2)

HR_combine <- paste0(hr,"(",HR.confint.lower, "~", HR.confint.upper, ")")

res<-c(co, HR_combine,hr, HR.confint.lower,HR.confint.upper,p.waldz, p.logrank)

names(res)<-c("Beta", "HR(95% CI)","HR","HR.confint.lower","HR.confint.upper", "Z_pval","Logrank_pval")

return(res)

})

results_f <- t(as.data.frame(mult_result_f, check.names = FALSE))

coxout_f <- as.data.frame(results_f)

coxout_f$gene = rownames(coxout_f)

write.table(coxout_f, file="cox_geneselection.csv", row.names = F, col.names = T, sep = ',', quote = F)

x<-coxph(Surv(PFS,PFS_status)~RB1+PIK3CA,data=mydat)

y<-summary(x)

y

#---------------------------------------------------------------------------------------------

### gene analysis related to ORR ###

mydat<-read.xlsx("",sheet=3,colNames = T)

result<-c()

for (i in (5:ncol(mydat))){ # i start fisher test

newdat1<-mydat[mydat$ORR==1,] #define groupA

GroupA_mut<-sum(newdat1[,i])

GroupA_wt<-nrow(newdat1)-GroupA_mut

newdat2<-mydat[mydat$ORR==0,] #define groupB

GroupB_mut<-sum(newdat2[,i])

GroupB_wt<-nrow(newdat2)-GroupB_mut

newdat<-c(GroupA_mut,GroupA_wt,GroupB_mut,GroupB_wt)

test<-matrix(newdat,nrow=2,ncol=2,byrow=TRUE)

qq<-fisher.test(test) #fisher.test() chisq.test

Odds_ratio<-qq[["estimate"]][["odds ratio"]] #check

p.result<-qq$p.value

result.linshi<-cbind(i,GroupA_mut,GroupA_wt,GroupB_mut,GroupB_wt,Odds_ratio,p.result)

result<-rbind(result,result.linshi)

}

linshi<-c(1:4) #### attention 1:q q=i-1

gene<-names(mydat[,-linshi]) ##??ȡ????????Ӧ??row.names(mydat)

result<-cbind(gene,result)

write.table(result, file="ORR.wff.csv",col.names = T,row.names = F,sep=",",quote=FALSE)

#--------------------------------------------------------------------------------------------------

# CIS analysis

library('stringr')

library('openxlsx')

library("survival")

library("survminer")

require("survminer")

######## data input #######

mydat<-read.xlsx("",sheet=2,colNames = T)

cutpoint<-read.xlsx("",sheet=3,colNames = T)

######cutoff value#####

cutoff<-cutpoint$CIS

##### regression analysis####

cox_regression <-c()

for (i in cutoff){

Group<-ifelse(mydat$CIS >= i,1,0)

mydat$Group<-Group

x <- coxph(Surv(PFS,PFS_status)~Group,data=mydat, na.action=na.exclude)

x <- summary(x)

uni <- as.data.frame(coef(x))

HR <- signif(x$coefficients[, "exp(coef)"], digits = 3)

HR.CI.lower <- signif(x$conf.int[,"lower .95"], digits =3)

HR.CI.upper <- signif(x$conf.int[,"upper .95"], digits =3)

HR <- paste0(HR,"(",HR.CI.lower, "-", HR.CI.upper, ")")

uni$HR<-HR

uni$HR.CI.lower<-HR.CI.lower

uni$HR.CI.upper<-HR.CI.upper

uni$TMB <- i

#combine

cox_regression[[i]]<-uni

}

########

cox_result <- do.call(rbind,cox_regression)

write.table(cox_result,file="result.csv",col.names = T,row.names = F,sep=",")

#----------------------------------------------------------------------------------------------

################ RB1 co-mutations ################

rm(list=ls())

file_path <- ""

patient_file <- ''

col_number <- 15

# parameters

###########set significant p value#############

pvalue = c(0.01,0.05)

pAdjMethod = "fdr"

eventFont = 4

eventSize = 0.74

legendBarHeight = 0.38

legendBarWidth = 0.73

##

nShiftSymbols = 5

sigSymbolColor = "black"

sigSymbolsSize = 1.7

sigSymbolsFontSize = 0.9

sigSymbolsFont = 3

#

pvSymbols = c(46,42)

limitColorBreaks = TRUE

nShiftSymbolsYAxis = 0.65

nShiftSymbolsXAxis = 5

library(RColorBrewer)

library(openxlsx)

setwd(file_path)

mydat<-read.xlsx( patient_file,colNames = T, sheet = 1)

mutMat<-mydat[,c(col_number:ncol(mydat))]

#pairwise fisher test source code borrowed from: https://www.nature.com/articles/ncomms6901

interactions = sapply(1:ncol(mutMat), function(i) sapply(1:ncol(mutMat), function(j) {f<- try(fisher.test(mutMat[,i], mutMat[,j]), silent=TRUE); if(class(f)=="try-error") NA else f$p.val} ))

# adjust p:

interactions[] <- p.adjust(interactions, method = pAdjMethod )

interactions = sapply(1:ncol(mutMat), function(i) sapply(1:ncol(mutMat), function(j) {f<- try(fisher.test(mutMat[,i], mutMat[,j]), silent=TRUE); if(class(f)=="try-error") NA else ifelse(f$estimate>1, -log10(interactions[i,j]),log10(interactions[i,j]))} ))

interactions

oddsRatio <- oddsGenes <- sapply(1:ncol(mutMat), function(i) sapply(1:ncol(mutMat), function(j) {f<- try(fisher.test(mutMat[,i], mutMat[,j]), silent=TRUE); if(class(f)=="try-error") f=NA else f$estimate} ))

rownames(interactions) = colnames(interactions) = rownames(oddsRatio) = colnames(oddsRatio) = colnames(mutMat)

sigPairs = which(x = 10^-abs(interactions) < 1, arr.ind = TRUE)

sigPairs2 = which(x = 10^-abs(interactions) >= 1, arr.ind = TRUE)

if(nrow(sigPairs) < 1){

stop("No meaningful interactions found.")

}

sigPairs = rbind(sigPairs, sigPairs2)

sigPairsTbl = data.table::rbindlist(

lapply(X = seq_along(1:nrow(sigPairs)), function(i) {

x = sigPairs[i,]

g1 = rownames(interactions[x[1], x[2], drop = FALSE])

g2 = colnames(interactions[x[1], x[2], drop = FALSE])

tbl = as.data.frame(table(apply(X = mutMat[,c(g1, g2), drop = FALSE], 1, paste, collapse = "")))

combn = data.frame(t(tbl$Freq))

colnames(combn) = tbl$Var1

pval = 10^-abs(interactions[x[1], x[2]])

fest = oddsRatio[x[1], x[2]]

d = data.table::data.table(gene1 = g1,

gene2 = g2,

pValue = pval, oddsRatio = fest)

d = cbind(d, combn)

d

}), fill = TRUE)

sigPairsTbl = sigPairsTbl[!gene1 == gene2] #Remove doagonal elements

sigPairsTbl[is.na(sigPairsTbl)] = 0

sigPairsTbl$Event = ifelse(test = sigPairsTbl$oddsRatio > 1, yes = "Co_Occurence", no = "Mutually_Exclusive")

sigPairsTbl$pair = apply(X = sigPairsTbl[,.(gene1, gene2)], MARGIN = 1, FUN = function(x) paste(sort(unique(x)), collapse = ", "))

sigPairsTbl[,event_ratio := `01`+`10`]

sigPairsTbl[,event_ratio := paste0(`11`, '/', event_ratio)]

sigPairsTblSig = sigPairsTbl[order(as.numeric(pValue))][!duplicated(pair)]

# PLOT

diag(interactions) <- 0

m <- nrow(interactions)

n <- ncol(interactions)

col_pal = RColorBrewer::brewer.pal(9, "PiYG")

col_pal = grDevices::colorRampPalette(colors = col_pal)

col_pal = col_pal(m*n-1)

interactions[lower.tri(x = interactions, diag = TRUE)] = NA

par(bty="n", mar = c(1, 4, 4, 2)+.1, las=2, fig = c(0, 1, 0, 1))

# adjust breaks for colors according to predefined legend values

breaks = NA

if(limitColorBreaks){

minLog10pval = 3

breaks <- seq(-minLog10pval,minLog10pval,length.out=m*n+1)

#replace extreme values with the predefined minLog10pval values (and avoid white colored squares)

interactions4plot = interactions

interactions4plot[interactions4plot < (-minLog10pval)] = -minLog10pval

interactions4plot[interactions4plot > minLog10pval] = minLog10pval

interactions = interactions4plot

}

#main plot

image(x=1:n, y=1:m, interactions, col = col_pal, # MAIN PLOT

xaxt="n", yaxt="n",

xlab="",ylab="", xlim=c(0, n+1), ylim=c(0, n+1),

breaks = seq(-3, 3, length.out = (nrow(interactions) * ncol(interactions))))

abline(h=0:n+.5, col="white", lwd=.5)

abline(v=0:n+.5, col="white", lwd=.5)

#events text

mtext(side = 2, at = 1:m, text = rownames(interactions), cex = eventSize,

font = eventFont)

mtext(side = 3, at = 1:n, text = rownames(interactions), cex = eventSize,

font = eventFont)

# add points

w = arrayInd(which(10^-abs(interactions) < min(pvalue)), rep(m,2))

points(w, pch=pvSymbols[2], col=sigSymbolColor, cex = sigSymbolsSize)

w = arrayInd(which((10^-abs(interactions) < max(pvalue)) & (10^-abs(interactions) > min(pvalue))), rep(m,2))

points(w, pch=pvSymbols[1], col=sigSymbolColor, cex = sigSymbolsSize)

#add p-value legends

points(x = n-nShiftSymbols, y = 0.7*n, pch = pvSymbols[2], cex = sigSymbolsSize) # "*"

text(x = n-nShiftSymbols, y = 0.7*n, paste0(" P < ", min(pvalue)), pos=4, cex = sigSymbolsFontSize, adj = 0, font = sigSymbolsFont)

points(x = n-nShiftSymbols, y = 0.65*n, pch = pvSymbols[1], cex = sigSymbolsSize) # "."

text(x = n-nShiftSymbols, y = 0.65*n, paste0(" P < ", max(pvalue)), pos=4, cex = sigSymbolsFontSize, font = sigSymbolsFont)

par(fig = c(legendBarHeight, legendBarWidth, 0, legendBarHeight), new = TRUE)

image(

x = c(0.75, 1),

y = seq(0, 1, length.out = 200),

z = matrix(seq(0,1,length.out = 200), nrow = 1),

col = col_pal, xlim = c(0, 1), ylim = c(0, 1), axes = FALSE, xlab = NA, ylab = NA

)

atLims = seq(0, 1, length.out = 7)

axis(side = 4, at = atLims, tcl=-.15, labels =c("> 3 (Mutually exclusive)", 2, 1, 0, 1, 2, ">3 (Co-occurence)"), lwd=.5, cex.axis = sigSymbolsFontSize, line = 0.2, font = sigSymbolsFont)

text(x = 0.4, y = 0.5, labels = "-log10 (p value)", srt = 90, cex = sigSymbolsFontSize, xpd = TRUE, font = sigSymbolsFont)

#-----------------------------------------------------------------------------------

### oncoplpt

setwd("")

library(stringr)

library(openxlsx)

library(ComplexHeatmap)

library(circlize)

library(colorspace)

library(GetoptLong)

#########data input############

clinical<-read.xlsx("",sheet=1,colNames = T)

mutation<-read.xlsx("",sheet=2,colNames = T,rowNames = T)

order<-read.xlsx("",sheet=3,colNames = T)

#making top annotation

top <- clinical[, c("Sex","Age","Smoking_history","Cancer_type",

"Stage","Treatment_line",

"ORR")]

top <- data.frame(t(top))

names(top) = clinical$ID

top<-rbind(top, mutation)

names(top) = clinical$ID

################# order#################

RO<-order$order

col_new = c("male"="#8b3e2f","female"="#000000",

"younger"="#63b8ff","older"="#1c9099",

"yes"="#984807","no"="#93cdd3","unknown"="grey",

"adc"="#c6ffd9","scc"="#17375e","nsclc"="#cb8bb0",

"III"="#3a3a31","IV"="#baba9c",

"na茂ve"="#948a54","treated"="#4a452a",

"PR"="#cb8bb0","SD"="#8bb0cb","PD"="black",

##############mutation type

"missense" = "#3cb371",

"inframe_indel" = "#6495ef",

"frameshift" = "#a020f0",

"CNV"="#8db40f",

"splicing"="#cd96cd",

"stop_gained"="#8b3a3a",

"Fusion"="#f0803a"

)

alter_fun = list(

background = function(x, y, w, h) {

grid.rect(x, y, w-unit(0.5, "mm"), h-unit(0.5, "mm"), gp = gpar(fill = "#CCCCCC", col = NA))

},

male = function(x, y, w, h) {

grid.rect(x, y, w-unit(0.5, "mm"), h-unit(0.5, "mm"), gp = gpar(fill = col_new["male"], col = NA))

},

female = function(x, y, w, h) {

grid.rect(x, y, w-unit(0.5, "mm"), h-unit(0.5, "mm"), gp = gpar(fill = col_new["female"], col = NA))

},

younger = function(x, y, w, h) {

grid.rect(x, y, w-unit(0.5, "mm"), h-unit(0.5, "mm"), gp = gpar(fill = col_new["younger"], col = NA))

},

older = function(x, y, w, h) {

grid.rect(x, y, w-unit(0.5, "mm"), h-unit(0.5, "mm"), gp = gpar(fill = col_new["older"], col = NA))

},

yes = function(x, y, w, h) {

grid.rect(x, y, w-unit(0.5, "mm"), h-unit(0.5, "mm"), gp = gpar(fill = col_new["yes"], col = NA))

},

no = function(x, y, w, h) {

grid.rect(x, y, w-unit(0.5, "mm"), h-unit(0.5, "mm"), gp = gpar(fill = col_new["no"], col = NA))

},

unknown = function(x, y, w, h) {

grid.rect(x, y, w-unit(0.5, "mm"), h-unit(0.5, "mm"), gp = gpar(fill = col_new["unknown"], col = NA))

},

adc = function(x, y, w, h) {

grid.rect(x, y, w-unit(0.5, "mm"), h-unit(0.5, "mm"), gp = gpar(fill = col_new["adc"], col = NA))

},

scc = function(x, y, w, h) {

grid.rect(x, y, w-unit(0.5, "mm"), h-unit(0.5, "mm"), gp = gpar(fill = col_new["scc"], col = NA))

},

nsclc = function(x, y, w, h) {

grid.rect(x, y, w-unit(0.5, "mm"), h-unit(0.5, "mm"), gp = gpar(fill = col_new["nsclc"], col = NA))

},

III = function(x, y, w, h) {

grid.rect(x, y, w-unit(0.5, "mm"), h-unit(0.5, "mm"), gp = gpar(fill = col_new["III"], col = NA))

},

IV = function(x, y, w, h) {

grid.rect(x, y, w-unit(0.5, "mm"), h-unit(0.5, "mm"), gp = gpar(fill = col_new["IV"], col = NA))

},

na茂ve = function(x, y, w, h) {

grid.rect(x, y, w-unit(0.5, "mm"), h-unit(0.5, "mm"), gp = gpar(fill = col_new["na茂ve"], col = NA))

},

treated= function(x, y, w, h) {

grid.rect(x, y, w-unit(0.5, "mm"), h-unit(0.5, "mm"), gp = gpar(fill = col_new["ntreated"], col = NA))

},

PR = function(x, y, w, h) {

grid.rect(x, y, w-unit(0.5, "mm"), h-unit(0.5, "mm"), gp = gpar(fill = col_new["PR"], col = NA))

},

SD = function(x, y, w, h) {

grid.rect(x, y, w-unit(0.5, "mm"), h-unit(0.5, "mm"), gp = gpar(fill = col_new["SD"], col = NA))

},

PD = function(x, y, w, h) {

grid.rect(x, y, w-unit(0.5, "mm"), h-unit(0.5, "mm"), gp = gpar(fill = col_new["PD"], col = NA))

},

#----------------------------------------------------------------------------------------------------

###################mutation types ####################################################

missense = function(x, y, w, h) {

grid.rect(x, y, w-unit(0.5, "mm"), h*0.8, gp = gpar(fill = col_new["missense"], col = NA))

},

inframe_indel = function(x, y, w, h) {

grid.rect(x, y, w-unit(0.5, "mm"), h*0.7, gp = gpar(fill = col_new["inframe_indel"], col = NA))

},

frameshift = function(x, y, w, h) {

grid.rect(x, y, w-unit(0.5, "mm"), h*0.6, gp = gpar(fill = col_new["frameshift"], col = NA))

},

CNV = function(x, y, w, h) {

grid.rect(x, y, w-unit(0.5, "mm"), h*0.6, gp = gpar(fill = col_new["CNV"], col = NA))

},

splicing= function(x, y, w, h) {

grid.rect(x, y, w-unit(0.5, "mm"), h*0.5, gp = gpar(fill = col_new["splicing"], col = NA))

},

stop_gained = function(x, y, w, h) {

grid.rect(x, y, w-unit(0.5, "mm"), h*0.4, gp = gpar(fill = col_new["stop_gained"], col = NA))

},

Fusion = function(x, y, w, h) {

grid.rect(x, y, w-unit(0.5, "mm"), h*0.3, gp = gpar(fill = col_new["Fusion"], col = NA))

},

large_fragment_indel = function(x, y, w, h) {

grid.rect(x, y, w-unit(0.5, "mm"), h*0.2, gp = gpar(fill = col_new["large_fragment_indel"], col = NA))

},

upstream_gene_variant = function(x, y, w, h) {

grid.rect(x, y, w-unit(0.5, "mm"), h*0.9, gp = gpar(fill = col_new["upstream_gene_variant"], col = NA))

}

)

pdf("onco.pdf", width = 13, height= 8)

oncoPrint(top, get_type = function(x) strsplit(x, ";")[[1]],

alter_fun = alter_fun, col = col_new,

column_order = names(top),

row_order = RO,

show_row_names = TRUE,

show_column_names = F,

# row_names_side = "left", pct_side = "right",

heatmap_legend_param = list(title = "Alternations",

at = c("male","female",

"younger","older",

"yes","no","unknown",

"adc","scc","nsclc",

"III","IV",

"na茂ve","treated",

"PR","SD","PD",

##############mutation type

"missense" ,

"inframe_indel" ,

"frameshift" ,

"CNV",

"splicing",

"stop_gained",

"Fusion",

"upstream_gene_variant",

"large_fragment_indel"),

labels = c("male","female",

"younger","older",

"yes","no","unknown",

"adc","scc","nsclc",

"III","IV",

"na茂ve","treated",

"PR","SD","PD",

##############mutation type

"missense" ,

"inframe_indel" ,

"frameshift" ,

"CNV",

"splicing",

"stop_gained",

"Fusion",

"upstream_gene_variant",

"large_fragment_indel")),

row_names_gp = gpar(fontsize = 10),

column_names_gp = gpar(fontsize = 10),

pct_gp = gpar(fontsize = 10)

)

dev.off()
